# Neurovascular Compression Syndromes of Cranial Nerves: A Multidisciplinary Guide to Management

**DOI:** 10.3390/brainsci16060569

**Published:** 2026-05-28

**Authors:** Madelyn Reilly, Nina Hashimoto, Kalvin Chen, Alan D. Kaye, Alaa Abd-Elsayed

**Affiliations:** 1 Anesthesiology Department, University of Wisconsin, Madison, WI 53706, USA; mjreilly2@wisc.edu (M.R.); nhashimoto2@wisc.edu (N.H.); kjchen7@wisc.edu (K.C.); 2 Department of Anesthesiology, Louisiana State University School of Medicine, Shreveport, LA 71103, USA; alan.kaye@lsuhs.edu

**Keywords:** neurovascular compression syndromes, cranial nerves, neurovascular conflict, cerebellopontine angle, trigeminal neuralgia, hemifacial spasm, glossopharyngeal neuralgia, microvascular decompression, stereotactic radiosurgery, neuromodulation, magnetic resonance imaging

## Abstract

**Highlights:**

**What are the main findings?**

- Neurovascular compression at the root entry zone, most commonly from arterial compression, drives focal demyelination, ephaptic transmission, and neuronal hyperexcitability in neurovascular compression syndromes, including trigeminal neuralgia, hemifacial spasm, and glossopharyngeal neuralgia.
- High-resolution MRI enables accurate diagnosis and surgical planning, while a stepwise management strategy, from pharmacotherapy to percutaneous procedures, stereotactic radiosurgery, microvascular decompression, and neuromodulation, optimizes patient outcomes.

**What are the implications of the main findings?**

- A multidisciplinary, anatomy-based approach improves diagnostic accuracy, individualizes treatment selection, and enhances long-term pain and functional outcomes.
- Emerging technologies, including endoscopic visualization, advanced neuromodulation, and virtual reality-assisted planning, have the potential to further refine surgical precision, reduce complications, and expand options for refractory patients.

**Abstract:**

**Background**: Neurovascular compression syndromes (NVCS) represent a spectrum of disabling neurologic disorders caused by vascular or structural compression of cranial nerves, most commonly at the root entry zone. Conditions such as trigeminal neuralgia (TN), hemifacial spasm (HFS), and glossopharyngeal neuralgia (GN) are associated with significant pain, functional impairment, and reduced quality of life. This review provides a multidisciplinary, anatomically grounded overview of the pathophysiology, diagnosis, imaging, and contemporary management strategies for NVCS. **Methods**: A narrative review of the literature was conducted, synthesizing historical perspectives, neuroanatomy of the cerebellopontine angle, mechanisms of neurovascular conflict, advances in imaging and neuromonitoring, and current treatment modalities. Medical, percutaneous, surgical, radiosurgical, and neuromodulatory approaches were evaluated, with emphasis on patient selection and outcome considerations. **Results**: Neurovascular compression, most frequently arterial compression at the root entry zone, leads to focal demyelination, ephaptic transmission, and neuronal hyperexcitability. High-resolution Magnetic resonance imagin (MRI) remains the diagnostic gold standard. First-line management for TN and related syndromes typically includes pharmacotherapy, particularly sodium channel blockers. Refractory cases may benefit from percutaneous rhizotomy, balloon compression, stereotactic radiosurgery, or microvascular decompression (MVD), which offers the most durable relief in appropriately selected patients. Emerging technologies, including endoscopic visualization, advanced neuromodulation, and virtual reality-assisted surgical planning, continue to refine treatment precision and safety. **Conclusions**: Effective management of NVCS requires a comprehensive understanding of neuroanatomy, pathogenesis, and individualized risk–benefit profiles. A multidisciplinary, stepwise approach optimizes outcomes and improves quality of life in patients with these complex disorders.

## 1. Introduction

Neurovascular compression syndromes (NVCS) are a group of disabling neurologic disorders caused by vascular or structural compression of cranial nerves (CN), most often at the root entry zone (REZ). Conditions such as trigeminal neuralgia (TN), hemifacial spasm (HFS), and glossopharyngeal neuralgia (GN) can produce severe pain, involuntary muscle contractions, and profound functional impairment that significantly diminish quality of life. These conditions, while individually uncommon, collectively represent a significant clinical burden due to their chronicity, impact on quality of life, and underlying mechanism of neurovascular compression, which directly informs both diagnosis and targeted treatment strategies. Although advances in imaging and microsurgical techniques have transformed care, optimal management remains complex and requires careful integration of anatomy, pathophysiology, and individualized treatment selection.

This review aims to provide a comprehensive, multidisciplinary guide to the diagnosis and management of NVCS. While there are various etiologies of NVCS, this article focuses on microvascular compression syndromes of CNs and their corresponding pathophysiology. It synthesizes current evidence on neurovascular conflict, advanced imaging modalities, medical therapy, percutaneous interventions, microvascular decompression (MVD), stereotactic radiosurgery (SRS), and emerging neuromodulation strategies. Given the evolution of high-resolution Magnetic Resonance Imaging (MRI) techniques and expanding minimally invasive and neuromodulatory treatment options, an updated synthesis of literature is necessary to guide clinicians in selecting the best evidence-based intervention. In addition, it highlights evolving technologies such as endoscopic visualization and virtual reality (VR) assisted surgical planning that are transforming contemporary practice.

Readers will benefit from this article by gaining a structured, anatomy-based framework for evaluating and treating these conditions. In addition to exploring practical insights into stepwise management strategies, readers will gain an updated overview of both established and emerging therapies. For clinicians across neurology, neurosurgery, pain medicine, anesthesiology, and dentistry, this review offers a concise yet comprehensive resource to support evidence-based decision-making and improve patient outcomes.

## 2. The Cranial Nerves

### 2.1. History of Cranial Nerve Compression Syndrome Management

While Galen, a Greek physician, originally described seven pairs of CNs based on animal dissections, the modern 12 CNs were first classified by the German anatomist Samuel Sommerring in 1778 [1]. However, the study of CNs and the syndromes associated with them has continued to evolve. Before the scientific understanding of nerves, treatments were based on general medical theories and observed symptoms. For example, the Persian physician Razi (865–925 CE) first described facial paralysis as a painless condition characterized by facial distortion and used treatments such as warming oils, special diets, herbal remedies, and bloodletting [2]. Additionally, John Fothergill provided the first complete and detailed description of TN in 1773 [3]. While early physicians acknowledged the intense pain associated with TN, the search for effective methods of pain relief remained inconclusive for centuries after numerous attempts at theories and ineffective treatment modalities.

Early neurosurgery for CN conditions emerged in the late 19th century. In 1891, British neurosurgeon Victor Horsley attempted the first intracranial operation for TN, which involved sectioning the preganglionic rootlets of the trigeminal nerve and was unfortunately fatal for the patient [4]. However, the first successful surgery was performed in 1892 by Fedor Krause, who sectioned two branches of the trigeminal nerve [5]. Since then, early surgical attempts have included neurectomy, decompression, and lesioning to destroy or disrupt affected nerve function, all of which are invasive and possess a significant risk of numbness or paralysis [6].

Modern scientific and technological advances have expanded the management of CN syndromes. This includes the emergence of microsurgical techniques, including Jannetta’s MVD. Developed by Dr. Peter Jannetta in 1966, MVD was based on the concept that pulsatile vascular compression at the REZ represents the primary mechanism underlying cranial nerve hyperactivity syndromes. In his seminal work, Jannetta demonstrated that surgical repositioning of an offending vascular structure could relieve symptoms without damaging the nerve, thereby establishing the neurovascular compression hypothesis and shifting away from destructive procedures toward etiologic surgical treatment [7]. Subsequent clinical experience confirmed that this approach was effective not only in treating TN, but also in treating HFS and GN. This conceptual framework remains the foundation of modern understanding of NVCS and continues to guide both diagnostic evaluation and surgical management. Minimally invasive techniques, such as SRS and endoscopic surgery, have since been explored to treat CN syndromes caused by neurovascular compression or tumors. SRS delivers focused beams of radiation to CNs or tumors and works by damaging the DNA of targeted cells, thereby halting their growth and activity [8]. The first SRS procedure was specifically performed for a patient with TN. In comparison, endoscopic procedures insert endoscopes through the nose (endonasal) or through a tiny incision behind the ear (retromastoid) to visualize compression points and either separate the nerve and vessel or remove tumors [9].

While these procedures have provided relief to some patients suffering from NVCS, many avoid surgical intervention and opt for medical management. While not a first-line treatment, botulinum toxin (BTX) injections, which inhibit the release of pain-causing neurotransmitters such as glutamate, can provide relief for patients who are refractory to oral medication [10]. BTX is a viable option for various forms of neuralgia, yet there is still a need for additional research for specific cases of neuralgia for proper dosing and treatment protocol. Additionally, anticonvulsants, such as carbamazepine and oxcarbazepine, target neuropathic pain by reducing the excitability of voltage-gated sodium channels in nerves and are particularly useful in treating trigeminal nerve disorders [11]. While there is also evidence that lamotrigine is being used specifically in the treatment of TN, more research is warranted. Furthermore, management of CN syndromes has shifted from a symptom-based approach to exploring and treating the underlying causes for lasting relief and improved quality of life.

### 2.2. Anatomical Nuances of Cranial Nerves and Related Vascular Structures of the Cerebello-Pontine Angle

**Figure 1:**
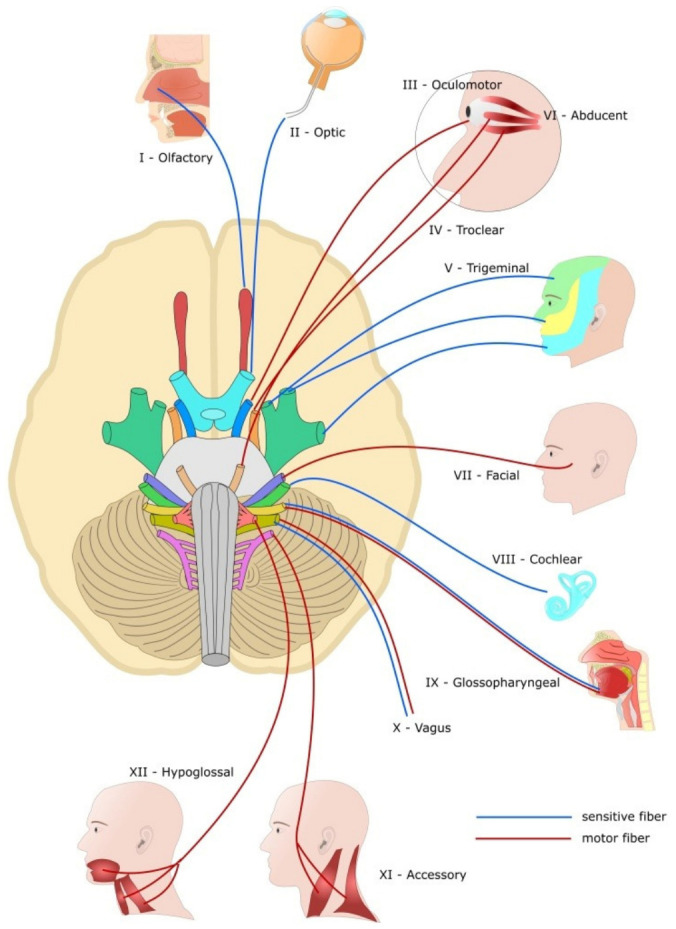
Anatomical depiction of the CPA cistern consisting of CN V, VI, VII, VIII, IX, X, and XI, as well as cranial nerves I, II, III, IV, and XII. (Reprinted/adapted with permission from Ref. [14] Sonne et al. 2025. This figure is published under the Creative Commons Attribution–NonCommercial–NoDerivatives 4.0 International License (CC BY-NC-ND 4.0), which permits noncommercial use, distribution, and reproduction in any medium, provided the original authors and source are credited).

The cerebellopontine angle (CPA) is an anatomically and clinically significant landmark in discussing the CNs and the compression syndromes associated with them. The CPA is a triangular region in the posterior cranial fossa that is bordered by the tentorium superiorly, the brainstem posteromedially, and the petrous part of the temporal bone posterolaterally [12]. Within the CPA is the CPA cistern, a subarachnoid space filled with cerebrospinal fluid (CSF) that contains CNs V, VI, VII, VIII, IX, X, and XI as well as blood vessels of the vertebrobasilar system, including the anterior inferior cerebellar artery (AICA) and the superior cerebellar artery (SCA) [13]. As shown in Figure 1, the anatomical distribution of CNs determines their innervation [14]. Lesions in this area can compress CNs, leading to a variety of neurological symptoms depending on the nerve involved.

CN V, the trigeminal nerve, is one of the most common nerves involved in NVCS and can progress to TN. CN V has three major divisions with different exit routes from the skull: V1 (ophthalmic nerve) exits through the superior orbital fissure into the orbit, V2 (maxillary nerve) exits through the foramen rotundum into the pterygopalatine fossa, and V3 (mandibular nerve) exits through the foramen ovale. CN VI, the abducens nerve, arises from the pontomedullary junction and passes through the cavernous sinus to enter the orbit via the superior orbital fissure. CN VII, the facial nerve, emerges from the pontomedullary junction and enters the petrous part of the temporal bone via the internal acoustic meatus, where it continues through the facial canal and exits the skull at the stylomastoid foramen. CN VIII, the vestibulocochlear nerve, emerges from the CPA and exits the posterior cranial fossa through the internal acoustic meatus. CN IX, the glossopharyngeal nerve, and CN X, the vagus nerve, travel through the CPA cistern before exiting the skull through the jugular foramen. In comparison, CN XII, the hypoglossal nerve, enters the premedullary cistern and leaves the cranial cavity through the hypoglossal canal [15].

Within the CPA, the CNs are closely exposed to numerous clinically relevant blood vessels. The AICA originates from the basilar artery, travels through the middle of the CPA, and has a close relationship with CNs VI, VII, and VIII. Compression of these CNs by the AICA can lead to HFS, sensorineural hearing loss, and abducens nerve palsy. The posterior inferior cerebellar artery (PICA) is a branch of the vertebral artery and is the most common artery to compress the lower CNs in the CPA. Compression of CN IX, X, or XI can cause GN or hemi-laryngopharyngeal spasm [16]. Additionally, the SCA, which originates from the terminal basilar artery in the upper CPA, can compress CN V and cause TN [17]. Because compression of the nerve by a blood vessel is a common cause of NVCS, understanding the anatomy of the nerves and vessels in the CPA is essential to diagnosing and treating these conditions. Furthermore, the hypoglossal nerve does not traverse the CPA cistern; conditions typically affected by CPA lesions or vascular compression do not affect CN XII.

Variations in the anatomy of the motor cranial nerves REZ, which is where a nerve enters or exits the brainstem or spinal cord, significantly affect the vulnerability of CNs to compression. The transition zone, which is the length of the nerve rootlet where the myelin sheath transitions from the tougher central nervous system (CNS) myelin produced by oligodendrocytes to the more delicate peripheral nervous system (PNS) myelin produced by Schwann cells, suggests that the CNS portion of the nerve is more vulnerable to pulsatile vascular compression than the PNS segment [18]. While the REZ and transition zones are often related and may overlap anatomically, they are not fully identical, as the transition zone is the actual vulnerable site where the transition of myelin takes place, which sits adjacent to or slightly distal to the REZ. Additionally, the exact location and length of this vulnerable transition zone vary among different CNs, which explains why specific nerves, including the trigeminal nerve, are more prone to compression syndromes [19]. Thus, individualized surgical approaches, nerve mapping, and a preoperative MRI are essential in MVD procedures to exclude structural causes of symptoms, minimize complications, and improve outcomes.

### 2.3. Pathogenesis of Neurovascular Compression in Cranial Nerve Syndromes

NVCS most commonly occurs from mechanical compression of the nerve by a blood vessel at a transition zone. With aging, arteries undergo vascular aging, a process characterized by elongation, stiffening, and loss of vascular elasticity [20]. Because of this phenomenon, vascular loop compression can occur, in which a blood vessel abnormally loops and compresses a CN [21]. Over time, chronic compression and pulsatile pressure lead to demyelination of axons, particularly in the transition zone where central and peripheral myelin meet [18]. This compression-induced demyelination leads to abnormal signaling and neuronal hyperexcitability that specifically underlies the paroxysmal pain seen in TN and related NVCS [21].

This mechanistic model of focal demyelination secondary to pulsatile vascular compression was originally proposed in the foundational work of Jannetta and has since been supported and refined by large anatomical-surgical series and modern imaging-based investigations [22,23]. In particular, anatomical studies have demonstrated that clinically significant neurovascular conflicts are most often located at the transition zone where central and peripheral myelin transition, while imaging studies have further shown that the severity and morphology of compression correlate with both symptom generation and surgical outcomes. These findings reinforce the concept that TN and related NVCS are not simply the result of neurovascular contact, but rather of structurally significant compression producing focal injury to the nerve.

However, contemporary TN literature indicates that simple neurovascular contact is not equivalent to clinically significant compression. Imagining-surgical correlation studies have shown that high-grade neurovascular conflicts producing morphological changes in the trigeminal root, such as displacement, distortion, or indentation, are more strongly associated with symptomatic TN than low-grade contact alone [22,24]. Furthermore, MRI has been shown to be more reliable for identifying severe conflicts than mild contact, and these higher-grade conflicts correlate strongly with favorable outcomes after MVD [22,25]. Accordingly, structurally significant neurovascular compression, rather than vessel-nerve contact alone, is considered the primary substrate underlying clinically relevant disease [24].

At a microscopic level, the primary driving force of alterations in the electrical activity of the brain is the demyelination-remyelination cycle. Initially, chronic compression leads to focal demyelination of a specific CN. To repair the damage, the body initiates remyelination by oligodendrocytes in the CNS and Schwann cells in the PNS. However, this repair is often insufficient or flawed, resulting in a thin, abnormal myelin sheath [26]. As a result, the cycle of demyelination and remyelination continues, which exacerbates the damage and compromises the nerve function. Ineffective myelination also results in ectopic impulse generation from ephaptic transmission as well as hyperexcitability and afterdischarges. Ephaptic transmission, otherwise known as cross-talk, occurs with insufficient myelination when an electrical activity from a high-frequency impulse in one nerve fiber can spread to and excite a nearby demyelinated fiber, generating a new and unintended impulse. Similarly, changes in ion channels in demyelinated axons make them more susceptible to spontaneous firing, in which an action potential can be generated without an external stimulus, as well as to afterdischarges, prolonged, self-sustaining bursts of neural activity that persist after a stimulus has stopped [27]. The Neurovascular Conflict Theory also proposes how pulsatile arterial compression at the REZ exacerbates the demyelination-remyelination cycle and resultant abnormal electrical signaling. Pulsatile blood flow through arteries near the brain and brainstem, corresponding to the heartbeat, leads to rhythmic compression of a CN. Because of the sensitivity of the REZ to trauma and irritation, repetitive pulsations against this area lead to microtrauma and further demyelination [28]. As a result, patients can experience stabbing pains, tingling, numbness, and spasms in regions innervated by the culprit nerve.

Superimposed on the demyelination-remyelination cycle that directly causes abnormal electrical activities, certain voltage-gated sodium channel subtypes undergo demyelination-induced remodeling that further contributes to neuronal hyperexcitability. In demyelinating diseases and injuries, ectopic clustering of sodium channels has been observed, where channels that are normally clustered around the nodes of Ranvier abnormally redistribute and pathologically accumulate at abnormal sites. Additionally, increased expression of sodium channels NaV1.7 and NaV1.8 has been reported in patients with inflammatory and neuropathic pain [29]. Such changes at the lesion site lower the depolarization threshold and promote axonal hyperexcitability. These findings are clinically relevant, as Nav1.7 and Nav1.8 are known to be central players of nociception signaling; in fact, sodium channel blockers are the foundation of pharmacological therapy for trigeminal neuralgia and other related NVCS [29].

When demyelination and ectopic firing become persistent, they will eventually cause central sensitization, explaining the chronic and refractory pain cases that are often difficult to treat. Persistent afferent signaling to the brainstem and other pain-processing regions eventually leads to facilitated nociceptive processing, synaptic neuroplasticity in the nociceptive pathway, and hyperexcitability of nociceptive neurons, mediating and even amplifying peripheral pain stimuli [30]. It has been shown that, unlike paroxysmal pain, continuous chronic pain is less responsive to sodium channel blockers [30]. In other words, central sensitization involves a distinct pain mechanism and thus requires different therapeutic or interventional targets.

While primary causes of NVCS are typically idiopathic and involve an adjacent blood vessel, secondary causes are identifiable medical conditions or structural abnormalities that directly compress or damage the nerve. Secondary causes can be divided into neoplasms and structural abnormalities, and into inflammatory and autoimmune disorders, and infection. Tumors, vascular anomalies, aneurysms, cysts, and arteriovenous malformations can press on CNs as they exit the skull and irritate them. Additionally, disorders such as multiple sclerosis (MS), systemic lupus erythematosus (SLE), and vasculitis can cause an inflammatory response of nerves or vessels and can result in compression. Finally, both viral and bacterial infections of nerves or of the CSF can directly irritate and damage CNs [31]. Thus, refining differential diagnosis and identifying prognostic factors related to the pathogenesis of these syndromes enables improved imaging interpretation, tailored treatment plans, and the avoidance of unnecessary procedures.

### 2.4. Epidemiology of TN, HFS, and GN

Epidemiology varies significantly by syndrome, with TN being the most common, affecting 4–20 per 100,000 people annually. TN has a higher prevalence in women and often presents in the fifth or sixth decade of life. HFS has an incidence of 1 per 100,000 people per year and is more common in women [29]. GN accounts for a small percentage of all cranial neuralgias, with an incidence of approximately 0.5 per 100,000 people. However, it is often misdiagnosed as TN and affects men and women equally [32]. Etiological distribution also varies by the CN involved. For example, 80–90% of TN cases are due to vascular compression. A majority of cases of HFS are also a result of neurovascular compression, whereas GN is typically idiopathic.

While many risk factors contribute to the development of CN syndromes, age is the primary factor, with most individuals affected after age 50. Other significant risk factors include vascular diseases that damage blood vessels, such as hypertension, diabetes, and smoking, as well as certain anatomical features, such as a crowded posterior fossa, a small CPA, or scar tissue from head trauma [33]. Certain genetic conditions, such as hereditary neuropathy with liability to pressure palsies, predispose nerves to damage from chronic pressure and can also increase the risk of developing CN syndromes [21]. Furthermore, advances in imaging techniques have enabled earlier detection of CN syndromes, more accurate vessel identification, and refined surgical planning.

### 2.5. Cranial Nerve Imaging

Before looking at diagnostic imaging, clinicians should first establish suspicion for NVCS through careful history review and physical examination. The patient’s symptom pattern, pain distribution, triggers, and neurologic examination remain crucial to diagnosis, while imaging should be used to support the clinical impression. The primary diagnostic objectives of CN imaging are to identify and precisely localize the underlying cause of clinical symptoms, facilitate treatment planning, and monitor disease progression. Imaging is crucial to identifying underlying pathology, such as tumors or cysts, inflammatory conditions, vascular disorders, or traumatic injuries. In addition to ruling out secondary lesions, imaging aims to pinpoint the exact anatomical location of the neurovascular conflict by visualizing the compression of a CN by a vascular structure and identifying the offending agent [34]. From facilitating surgical planning to predicting treatment response, sophisticated imaging techniques have enabled advances in the diagnosis and management of NVCS.

While many imaging modalities are available for CN imaging, MRI is the gold standard due to its excellent soft-tissue contrast, which allows detailed visualization of structures within the brain as well as the nerves. The primary role of MRI is to exclude alternative structural causes, such as CPA tumors, epidermoid cysts, demyelinating lesions, vascular malformations, or aneurysms, and only secondarily supports the anatomical characterization of the suspected neurovascular conflict. Importantly, radiologic evidence of neurovascular contact must be interpreted within the broader clinical context, because vascular contact with CN can also be present in individuals without symptoms. Thus, MRI should strengthen or clarify a clinically suspected diagnosis rather than serve as the sole determinant of treatment. MRI machines visualize nerves using powerful magnetic fields and radio waves to detect signals from hydrogen atoms in the body’s water molecules, which are then converted into detailed cross-sectional images of the body’s soft tissues, including nerves [35]. Specifically, T2-weighted imaging is commonly used to visualize CNs because it highlights fluid, such as CSF, and shows nerves as distinct, dark structures against the bright CSF [36]. This contrast is particularly useful for identifying the cisternal segments of the nerve, where it is surrounded by CSF and most susceptible to vascular compression. High-resolution sequences, such as Constructive Interference in Steady States (CISS) and Fast Imaging Employing Steady State Acquisition (FIESTA), can further visualize small structures, including nerve segments, with great clarity [37]. These sequences provide high spatial resolution and CSF-tissue contrast, allowing precise delineation of the REZ, which is the most common site of compression. They also enhance the visualization of adjacent vascular loops, enabling the identification of offending vessels and the relationship to the nerve. Findings are particularly relevant when imaging shows not only contact, but also morphologic nerve changes such as displacement, indentation, distortion, or atrophy at the root entry or exit zone. Studies correlating MRI with intraoperative findings have demonstrated that MRI is most reliable for identifying high-grade neurovascular conflict, particularly when nerve displacement or indentation is present [22]. MRI grading should be incorporated into preoperative counseling and surgical selection rather than being treated as a purely descriptive radiologic finding. High-resolution MRI should not be the indication for intervention; it should support the differential diagnosis, anatomical characterization, and surgical planning.

Additionally, Computed Tomography (CT) can be used to visualize the bony canals and skull base landmarks through which the nerves pass, which are difficult to see on other scans. The use of CT often complements MRI and is valuable for evaluating the intraosseous segments of nerves, detecting bony lesions or trauma, and providing high-quality 2D and 3D multiplanar reconstructions to trace the complex course of nerves [38].

The use of MR angiography fused with 3D time-of-flight spoiled gradient-recalled sequence (3D TOF MRA) may allow for diagnosis of the offending vessels specifically for TN [39]. 3D TOF MRA sequence may show the fast-flowing blood, which gives excellent visualization of the arterial anatomy because the arteries appear hyperintense, contrasting with venous structures. 3D FIESTA is a high-resolution volumetric sequence, which can increase contrast between CSF and tissues. The CSF appears hyperintense with blood vessels hypointense, and the cranial nerves are isotense relative to the brain stem. These visualizations allow for the detection of neurovascular compression. The fusion of the 3D FIESTA and 3D TOF MRA allows for the great anatomical detail from the 3D FIESTA combined with 3D TOF MRA, which allows for easy and quick visualization of the neurovascular compression [40]. While MRA may give a comprehensive understanding of the neurovascular compression, it may also miss veins, small arteries, and arteriolar branches. A 3D-MFI approach may allow better precision in the preoperative planning of treatment for NVCS [41].

Though traditional imaging modalities have enabled practitioners to adequately visualize CNs, emerging techniques are being explored to overcome the significant limitations of conventional methods in visualizing these small, complex, and delicate structures and to provide more precise, detailed information for diagnosis, surgical planning, and intervention. One such technique is Neuromuscular Ultrasound (NMUS), which can directly visualize specific, readily accessible nerves, such as the optic, facial, vagus, and spinal accessory nerves. Additionally, NMUS can indirectly evaluate other nerves by examining the muscles they innervate [42]. While this technique is safe, inexpensive, and often used as a complement to different studies, it currently has a limited scope. Diffusion sequences, such as Diffusion Tensor Imaging (DTI), are used to evaluate nerve fiber integrity by mapping the diffusion of water molecules along the nerve fibers. DTI tractography is most effective for the larger, more easily visualized CNs, such as CN II, III, V, VI, VII, and VIII. Still, it is less effective for the lower CNs, since their fibers are thinner and more diffuse [43]. While this technique creates a 3D reconstruction to visualize the nerve’s path, which is helpful for surgical planning to avoid damaging essential nerve fibers, it is often complemented by other sequences for a complete assessment.

In addition to the above-mentioned imaging techniques that allow for direct visualization of the target structures, other functional imaging modalities may provide complementary roles during the evaluation of NVCS. Though not currently used in routine clinical practice for NVCS, both DTI and functional MRI (fMRI) have shown emerging utility. FMRI specifically is useful for identifying pain-associated activation patterns in the central nervous system, including the insula, thalamus, and anterior cingulate cortex, as well as other cortical/subcortical areas. Studies have shown that TN patients have increased blood flow to the above-mentioned brain regions, suggesting the involvement of central pain-processing networks in NVCS beyond the peripheral nerves [44]. It has been suggested that high preoperative connectivity in the limbic system is predictive of better responses to surgical interventions such as percutaneous rhizotomy [45]. In other words, both DTI and fMRI may be useful in assessing the extent of nerve injuries and predicting surgical outcomes. On the contrary, PET-CT, which in general is used for characterizing metabolic activities, has been shown to have no established role in nerve injury assessment, surgical outcome prediction, and radiosurgery guidance [46]. While these functional imaging modalities are undergoing active investigation as promising tools for NVCS evaluation with some emerging evidence, they are not currently a part of routine use.

Radiologic patterns can vary by nerve, but each CN can exhibit similar radiologic patterns based on the anatomy and common pathologies associated with that nerve. CN I (olfactory) is associated with anterior skull base fractures on CT as well as olfactory groove masses with avid enhancement and possible bony destruction (neuroblastoma) [47]. CN II (optic) is associated with nerve swelling and enhancement on fat-suppressed T1 images (optic neuritis) and fusiform enlargement of the nerve or an enhancing mass within the optic canal (gliomas or meningiomas) [48]. CN III (oculomotor), IV (trochlear), and VI (abducens) are associated with lesions in the cavernous sinus or superior orbital fissure, appearing as an enhancing mass or inflammation affecting multiple nerves in that region, as well as aneurysms and denervation atrophy of extraocular muscles [49]. CN V (trigeminal) is associated with neurovascular compression at the REZ on high-resolution T2 MRI (neuralgia), an enhancing mass along the nerve course (tumor), and enlargement of the foramen ovale/rotundum [50]. CN VII (facial) is associated with nerve enhancement along the course in the temporal bone (Bell’s Palsy), an enhancing mass in the internal auditory canal (Schwannoma), and capsular loop compression of the nerve in the cisternal segment (spasm) [51]. CN VIII (vestibulocochlear) is associated with a well-defined, enhancing mass in the CPA or internal auditory canal (Schwannoma) [52]. CN IX (glossopharyngeal), X (vagus), and XI (accessory) are associated with an enhancing mass in the jugular foramen (Schwannoma or meningioma) [53]. CN XII (hypoglossal) is associated with lesions in the hypoglossal canal with enlargement of the canal and nerve enhancement or thickening [54]. While these entities are not primary examples of neurovascular compression, they are still critical considerations in the differential diagnosis of cranial nerve dysfunction, as many can mimic the clinical presentation of NVCS. Radiologic evaluation, therefore, plays a key role in not only identifying neurovascular conflict but also excluding alternative etiologies.

### 2.6. VR in Cranial Nerve Syndromes

VR-based technologies have recently been incorporated into the evaluation of CN syndromes as advanced tools for anatomical visualization and spatial analysis. 2D images play an essential role in building the 3D visualization models used for neurosurgical planning. In NVCS, these technologies are best viewed as adjunctive planning and educational tools rather than established methods proven to improve clinical outcomes. MRI is often preferred for its superior soft-tissue contrast, while CT can be used to visualize bony structures. T1-weighted MRIs can capture the skin’s topography, creating a surface map of external anatomical points. T1W and T2W MRI sequences can differentiate cerebral tissue layers. After alignment and calibration of the multimodal imaging, 3D visualization can be created, reflecting the true anatomical complexity of the skull base [55]. These patient-specific 3D visualizations are essential in NVCS such as TN, HFS, and GN, where the precise visualization of neurovascular compression at the REZ is critical for identifying the offending vessel and planning intervention. However, the current literature on VR and 3D image-based planning for MVD appears to be primarily focused on feasibility, anatomical visualization, and presurgical simulation, rather than providing high-level evidence of superior patient outcomes. The immersion and interaction allow the user within active stereoscopic glasses and controllers to have both interaction and aided immersion with haptic feedback. Modifications to address the glasses’ smaller field of view and the restricted work area have been made over time. The addition of user tracking has provided a seamless user-environment interface, and VR has been integrated into neurosurgery, with added importance. VR-based training models can help understand neuroanatomy using these systems [56]. As for MVD, VR allows surgeons to simulate the surgical approaches and plan for the patient-specific anatomy before surgery, potentially improving anatomical orientation and preoperative confidence. Similarly, 3D CT reconstruction assisted by augmented reality can be used for preoperative planning in CN syndromes. 3D reconstruction technology aims to enhance the accuracy of MVD. The integration of 3D-CT with augmented reality streamlines the surgical procedure, potentially minimizing uncertainty and complexity [57]. VR has recently been incorporated into the treatment and diagnosis of CN syndromes, enabling enhanced visualization of the surgical field and a better understanding of neuroanatomy.

## 3. Trigeminal Neuralgia (TN)

### 3.1. Diagnosis of TN and Trigeminal Neuralgia Mimics

TN is a relatively rare form of orofacial neuropathic pain. This condition presents with episodic severe pain in the face that is usually described as a shooting or stabbing pain. These episodes of pain may be triggered by non-nociceptive stimuli such as eating or speaking [58]. The trigeminal nerve (CN V) is the largest CN and is responsible for the detection of sensory stimuli arising from the craniofacial area [59]. TN most frequently involves the second or third divisions of the trigeminal nerve and is more common on the right side of the face; however, there are select bilateral cases of TN [60].

TN is categorized into classical TN (associated with neurovascular compression), secondary TN (due to underlying neurological disease, such as a tumor or MS), and idiopathic TN. This classification reflects the recognition of TN as a heterogeneous disorder with distinct clinical and pathophysiologic subtypes. Burchiel further refined this framework by distinguishing between purely paroxysmal (classical TN) with concomitant continuous pain (secondary TN), emphasizing differences in symptomatology, underlying mechanisms, and response to treatment [61]. The criteria for diagnosis of TN according to the ICHD-3 are: (1) recurrent paroxysms of unilateral facial pain in the distribution of the trigeminal nerve, electric shock-like, sharp, or shooting pain for a fraction of a second to 2 min with severe intensity, (2) innocuous stimuli within the affected nerve distribution precipitate the pain, and (3) no other better diagnosis. Classical TN is the most common form of TN [60,62]. Although no apparent underlying genetic association has been identified for idiopathic TN, it is thought to result from both peripheral and central mechanisms [63].

Compression of the trigeminal nerve, most commonly by an artery, is the primary cause of classical TN and may present as paroxysmal, provokable, unilateral, and not accompanied by sensory loss [64]. Subsequent nerve demyelination in the area can also lead to classical TN. Patients may also experience concomitant continuous pain that may be associated with trigeminal nerve root atrophy, suggesting that this pain stems from axonal loss and abnormal activity in denervated trigeminal second-order neurons [65]. It is essential to distinguish between classical TN and secondary TN due to the associated symptoms. Classical TN is associated with sudden shocks of focal pain that are short-lasting, while secondary TN is characterized as constant pain, though with lower intensity [66]. The distribution of pain in classical TN may also reflect the topography of neurovascular conflict along the trigeminal root. There is a correlation between the anatomical site of vascular compression and the peripheral distribution of pain, supporting a somatopic organization of the trigeminal sensory root [23]. Although this relationship is not true for all patients, it provides clinically relevant insight linking symptom localization to operative anatomy. Once TN is diagnosed, patients typically undergo an MRI to exclude other pathologies, such as tumors, that could cause secondary TN [67]. MRI is highly effective for identifying structural causes, but it cannot reliably distinguish TN from other facial pain syndromes without correlation to clinical presentation [68].

The orofacial region can present with multiple etiologies due to the sheer number of nerves in the area, including the trigeminal nerve, which can mimic TN symptoms. Hemicrania continua (HC) is a persistent unilateral headache with superimposed exacerbations of moderate to severe intensity. Although HC may be misdiagnosed as TN, it is a distinct primary headache disorder with different diagnostic criteria and treatment response. In one study, a patient developed headaches and daily facial pain two weeks after wisdom teeth extraction. Initially diagnosed with TN, the patient was hospitalized for further evaluation of his condition [69]. Myofascial pain dysfunction syndrome (MPDS) is a musculoskeletal condition characterized by regional muscle pain and trigger points. Unlike TN, which presents with brief, electric shock-like paroxysms, MPDS produces persistent, dull, and aching pain [70]. Because of overlapping signs and symptoms between MPDS and TN, misdiagnoses are common, and additional diagnostic testing is needed for proper management [71]. TN is a complex disorder that requires proper diagnosis and treatment for affected patients.

### 3.2. Psychological Aspects of Trigeminal Neuralgia

TN is associated with significant psychological comorbidities that warrant significant consideration during clinical evaluation and treatment. TN is considered a highly disabling condition due to its profound effects on emotional well-being and daily functioning. The psychological burden of TN has been closely linked to both the severity and unpredictability of pain episodes that contribute to the high rates of depression and anxiety in patients with TN [72]. TN with recurrent paroxysmal pain due to neurovascular compression can contribute to psychological comorbidities [71]. Patients with TN can often demonstrate a higher prevalence of depressive disorders, anxiety disorders, sleep disorders, fear of attacks, anticipatory avoidance, social withdrawal, and treatment-related distress. These effects are best understood as secondary consequences of chronic neurovascular compression-mediated pain rather than primary psychiatric pathology. This increased risk is thought to arise from a combination of chronic pain-induced stress responses and repeated activation of limbic and hypothalamic–pituitary–adrenal pathways. In addition, the sudden and severe nature of some TN pain episodes may increase the risk of psychiatric disorders due to fear conditions and maladaptive coping mechanisms [73]. Sleep-related symptoms are often attributed to pain-related awakenings and also show a positive correlation with pain intensity, suggesting that more severe pain contributes to worsening psychological symptoms [74].

Quality of life in patients with TN is often reduced, and the increased duration of pain has been associated with greater psychological impairment, including anxiety and depression [75]. These psychological effects contribute to the overall disease burden and functional limitations experienced by patients with TN [76]. Disability related to TN is common and often interferes with daily activities. In fact, a substantial portion of patients report prolonged periods of absence from work or social responsibilities, accompanied by depressive symptoms and negative thoughts [77]. Standardized psychological assessments such as the Hamilton Depression Rating Scale (HDRS) have demonstrated a positive association between depressive symptoms severity and disease duration, suggesting that there is a psychological impact of ongoing neuralgia [72,78].

Furthermore, greater involvement of multiple trigeminal branches and longer duration have been associated with increased depressive symptoms and mood disturbances. These factors likely originate from the pain increase and psychological distress through the stress-related neural pathways, further exacerbating depressive symptoms over time [79]. The importance of evaluating psychological conditions related to TN is needed as psychological conditions can lead to diminished quality of life and need to be addressed in the affected patients.

### 3.3. Trigeminal Neuralgia in a Dental Setting: The Necessity of Awareness

TN frequently presents first in dental care because of pain localized to the orofacial region and is often described as tooth pain. Classic triggers for this pain include chewing, speaking, and brushing teeth, so patients may first seek a dentist. It is essential to distinguish dental conditions from TN, and dentists play a crucial role in early recognition and in avoiding unnecessary procedures while ruling out temporomandibular disorders [80]. TN may present as tooth-localized pain, leading patients to initially seek dental evaluation [81]. However, odontogenic pain is typically continuous and associated with identifiable dental pathology, whereas TN is characterized by brief, stimulus-triggered paroxysms.

In comparison, a nonodontogenic toothache may be present with features that do not clearly show a single tooth origin. TN can present as a toothache, and early recognition of these distinguishing features is essential to avoid misdiagnosis and unnecessary procedures [82]. As such, a concerning number of unnecessary dental extractions occur with patients suffering from TN, many times at the request of the patient [83,84]. Delays in diagnosis are common when initial consultations occur outside neurology, and the misclassification of the complaint can postpone proper diagnosis and treatment for months. It is essential to raise awareness in dentistry and primary care to ensure timely recognition and treatment [85]. Because TN frequently presents as tooth-localized pain, dental clinicians should have a high index of suspicion to achieve an accurate diagnosis and promptly refer to the appropriate specialist for treatment.

### 3.4. Non-Surgical Management of TN

Non-surgical management represents the initial treatment option for most patients with TN, and most patients often begin with conservative management before proceeding with surgery. Pharmacotherapy remains the cornerstone of therapeutic management due to its minimally invasive nature and has been established as a first line of treatment. Antiepileptic and anticonvulsant medications are the foundation of pharmacotherapy, with carbamazepine remaining a first-line pharmacologic treatment for TN. The mechanism of action of the medication involves blocking the voltage-gated sodium channel, thereby reducing hyperexcitability in the trigeminal pathways [86]. Although carbamazepine is an effective treatment, side effects are common, including drowsiness, dizziness, rash, liver damage, and its long-term use has not been thoroughly studied [87,88].

An alternative to carbamazepine is oxcarbazepine, which is often an accepted second-line treatment. However, oxcarbazepine may also produce unwanted side effects, though not at the rate of carbamazepine [89]. Oxcarbazepine is the keto-analogue of carbamazepine that is rapidly converted into its pharmacologically active 10-monohydroxy metabolite. While carbamazepine can provide better pain relief, it may produce higher rates of unwanted side effects [90]. The side effects of oxcarbazepine include somnolence, headache, gait instability, hyponatremia, and thrombocytopenia. The most serious side effect of thrombocytopenia can be observed within one month of treatment and is often dose-dependent. Both carbamazepine and oxcarbazepine are often considered best-line therapies, with oxcarbazepine offering similar efficacy to carbamazepine with a better side effect profile.

For patients who cannot tolerate carbamazepine and oxcarbazepine or do not find adequate relief, alternative medications may be used. Baclofen is a GABAB receptor agonist that is used for patients who are unable to tolerate carbamazepine. Its therapeutic effect is thought to result from modulation of inhibitory neurotransmission within central pain pathways, providing benefit in select patients with refractory symptoms [91]. Intravenous lidocaine has also been found to be another non-surgical option for acute symptom management in TN. Lidocaine and its oral congeners exert sodium channel blockade in a dose-dependent manner in both the peripheral and central nervous systems. Its use has been shown to reduce short-term pain; however, long-term therapy is effective in select patients [92].

While supportive care may improve overall patient well-being, the primary focus of TN management in the context of NVCS remains targeted treatment of neurovascular conflict through pharmacologic, percutaneous, or surgical approaches [93]. As such, pharmacological therapy remains central to the management of TN, but patient-centered care also plays a vital role in improving patient quality of life.

### 3.5. Percutaneous Approaches: Radiofrequency and Glycerol Rhizotomy

Percutaneous approaches are often considered for patients with TN who do not experience relief from pharmacotherapy or are not candidates for surgical intervention. These techniques involve minimally invasive procedures that can disrupt pain-transmitting fibers in the trigeminal nerve. Radiofrequency rhizotomy (RFR) and glycerol rhizotomy (GR) are widely used, well-established percutaneous approaches.

RFR is a percutaneous technique that selectively thermocoagulates the trigeminal nerve fibers that are responsible for pain transmission. RFR is typically performed under fluoroscopic or CT guidance with electrode placement at the Gasserian ganglion [94]. The mechanism of RFR is strongly influenced by temperature-dependent differential injury to trigeminal nerve fibers. Radiofrequency energy produces controlled thermal lesions that preferentially affect small-diameter, pain-transmitting Aδ and C fibers. Compared with other available percutaneous methods, RFR has demonstrated short-term pain relief, although the long-term effects and recurrence vary from case to case [95]. In cases of recurrence, it is effective in patients following initial treatment [96]. Repeat RFR has been shown to provide comparable pain relief to initial procedures without a significant increase in complication rates, supporting its use in recurrent cases [97]. Long-term observational data further suggest that RFR targeting the Gasserian ganglion may provide sustained pain relief [98]. Despite the advantages of RFR, pain recurrence is still a recognized limitation and should be considered with the need for repeat procedures over time. In addition, the potential sensory implications of RFR should be discussed during preoperative counseling. Paresthesia appears to be more common after RFR than alternatives such as SRS [94]. Facial numbness is also influenced by procedural parameters, particularly lesioning temperature, with higher temperatures associated with an increased risk of postoperative numbness [95]. Other clinically relevant sensory complications include hypoesthesia and reduced corneal reflex, which may increase the risk of ocular morbidity [98]. These risks need to be considered when deciding on treatment options due to the nature of the procedure.

GR is an alternative percutaneous technique that involves injecting glycerol into the trigeminal cistern. Using CT, the needle tip can be guided from the foramen ovale to Meckel’s cave, and confirmation of the location can be obtained. To confirm, a trigeminal cisternography after cone-beam CT can be made, and glycerol (0.2–0.4 mL) can be injected into the trigeminal cistern. The treatment preferentially affects pain fibers while often preserving motor function and reflexes [99]. GR is commonly preferred in elderly patients and patients at higher risk for more invasive procedures. Initial pain relief from GR is rapid, and repeat injections may be considered if symptoms recur [100]. Similarly to RFR, pain relief varies, and long-term efficacy is lower than with MVD [101]. Comparative studies indicate that patients undergoing GR are older and have lower efficacy than those undergoing MVD [102]. Additionally, outcomes vary, with some reports finding limited sustained benefit in the long term [103]. When considering both RFR and GR, care should be taken to maximize treatment efficacy and limit recurrence.

### 3.6. Percutaneous Approaches: Balloon Compression

Percutaneous balloon compression (PBC) is a well-established minimally invasive technique for the treatment of TN for patients who are poor candidates for surgery. The procedure targets the trigeminal ganglion via controlled mechanical compression to disrupt pain-transmitting fibers while preserving function. PBC is typically performed by advancing a catheter through the foramen ovale under fluoroscopic or CT guidance. Current techniques incorporate Dyna-CT to optimize catheter trajectory, confirm accurate placement within Meckel’s cave, and assess balloon morphology and volume [104,105]. Once the correct position is confirmed, Omnipaque is injected into the catheter to inflate the balloon. A “pear-shaped” balloon often indicates that the balloon is positioned correctly within the trigeminal cistern. Balloon volume and inflation pressure are carefully controlled, and compression of the ganglion is maintained for several minutes before deflation and removal of the catheter [104,106]. The mechanical deformation of the ganglion results in focal demyelination and the interruption of abnormal pain-transmitting fibers, while preserving the sensory nerves. The duration of compression and the degree of balloon inflation are essential parameters to consider; excessive compression can lead to sensory loss, while inadequate compression may result in insufficient pain relief [106]. The procedure is typically performed under general anesthesia; however, conscious sedation and local anesthesia techniques may be feasible if the preservation of sensation is preferred or if general anesthesia imposes increased risks [107]. Compared with GR, balloon compression relies on mechanical rather than chemical neurolysis. GR can damage pain fibers with glycerol, whereas balloon compression produces a lesion by deforming the ganglion. GR may require fewer resources, but PBC can provide a more standardized and predictable lesion [108]. Balloon compression is another minimally invasive procedure for patients at risk for other procedures that has proven to be safe and effective as a treatment for TN.

### 3.7. MVD for TN: Surgical Pearls

MVD is a surgical option for patients with TN who do not receive adequate relief from pharmacological therapy and percutaneous approaches. Unlike percutaneous approaches, MVD aims to treat the underlying cause of TN by relieving vascular compression of the offending vessel on the trigeminal nerve without damaging nerve fibers [109]. MVD is performed under general anesthesia through a posterior fossa craniectomy, typically via a retrosigmoid approach. Through the opening, the cerebellum is retracted to expose the trigeminal nerve REZ. Careful analysis and dissection are made to identify the arteries or veins that are compressing the nerve. This distinction is clinically important because arterial and venous conflicts are not equivalent. Arterial compression is more commonly associated with classical TN and has been linked in several series to higher rates of durable pain relief following MVD, likely reflecting the greater force exerted by arterial structures at the REZ [110,111,112].

In contrast, venous compression represents a more heterogeneous entity with variable clinical presentation and surgical outcomes. Although acceptable results have been reported in select cases of venous compression, these cases often require more individualized intraoperative decision-making. Accordingly, operative planning should characterize whether the conflict is arterial, venous, or mixed and whether it produces clear morphological deformation of the nerve, rather than simply documenting the presence of neurovascular conflict. Once the offending vessel is identified, it is dissected off, and Teflon is interposed between the offending vessel and nerve to maintain separation. In cases where there is no improvement or recurrent symptoms, revision of the MVD may yield preferable results [109,113,114]. In cases where venous compression is present, veins may be carefully mobilized, divided, or padded, depending on their size, to avoid avulsion [114]. Attention to detail of the technique is critical in reducing the rate of complications. The use of adjunct materials, such as calcium phosphate cement, has been associated with reduced rates of CSF leaks [115]. Careful measures of careful dural closure and CSF management contribute to the long-term procedural success and lower complication rates [116]. Preoperative MRI plays a vital role in surgical planning and helps guide operative strategy. At the same time, postoperative MRI may be used to confirm decompression of the trigeminal nerve and to visualize the Teflon between the arteries and the nerve [117]. Furthermore, MVD is a promising treatment for patients, offering proven, lasting results with the potential for repeat treatment.

### 3.8. MVD for TN: Microscope vs. Exoscope vs. Endoscope

**Figure 2:**
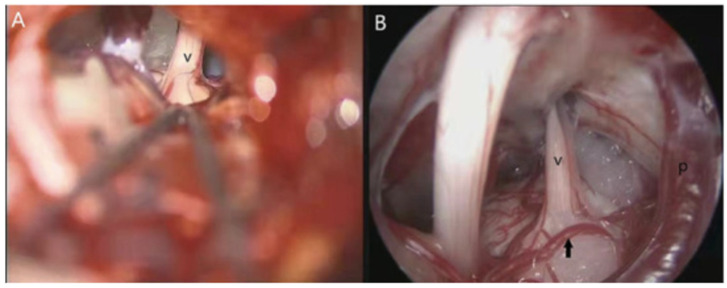
The same visual field (**A**) with a microscope and (**B**) with the use of an endoscope. v—trigeminal nerve, p—petrosal vein, and arrow showing the responsible compressing artery that is not found under the microscope. (Reprinted/adapted with permission from Ref. [118] Yu et al. 2022. This figure is distributed under the terms of the Creative Commons Attribution (CC BY) License, which permits unrestricted use, distribution, and reproduction in any medium, provided the original authors and source are credited).

The visualization technique plays a critical role in MVD for TN, as the procedure’s success depends on accurate identification of the trigeminal nerve and the offending vessels. The microscope has long been the standard for visualization; however, it offers a limited field of view and may limit visualization of vessels, making the operation challenging. The endoscope has seen increased use due to its improved illumination and broader field of view. The endoscope provides an angled visualization of the nerve REZ, allowing a clearer view of the compressing vessels than a microscope. This method also allows for enhanced visualization of Teflon placement between the nerve and vessel (Figure 2) [118]. Endoscopic techniques also provide improved access in confined spaces and may reduce the need for cerebellar retraction. Clinical outcomes with an endoscope are compared with those with a microscope, but the endoscope is superior for visualization of the operating field [119]. It has also been found that the use of an endoscope has yielded shorter operation times as well as lower complication rates, suggesting the superiority of the endoscope [120]. While both the microscope and endoscope are established methods, the newer exoscope combines high-definition digital imaging with external display systems. It offers high quality and allows surgeons to operate with better ergonomic positioning than traditional methods.

The exoscope provides the team with high-definition views of the surgical field, enabling its use for teaching and collaboration. It has been comparable in results to the microscope; however, the exoscope tends to overexpose the surgical field due to its wide-angle visualization [121,122]. Operative times using exoscopic approaches may be slightly longer than those reported with endoscopic and microscopic techniques, though the complication rates are lower [123]. As such, endoscopic approaches are widely adopted for their visualization benefits, whereas the exoscope is an evolving technology that may, with further development, provide comparable effectiveness while enhancing surgeon ergonomics and workflow.

### 3.9. MVD for TN: Decompression vs. Transposition

MVD is widely accepted as the standard treatment for TN once the compression is identified. For this procedure, two techniques are commonly used: decompression (interposition) and transposition. Both techniques aim to eliminate the compression at the nerve REZ but differ in how the offending vessel is managed. Decompression is the standard and most commonly used MVD technique. This technique carefully mobilizes the offending vessel away from the trigeminal nerve, and Teflon is then placed between the vessel and nerve to maintain separation. This technique is generally preferred because it is straightforward and associated with a lower risk of long-term complications. It is usually sufficient in cases where the offending vessel is small and can be displaced without significant tension [124]. However, decompression may introduce risks such as strokes, postoperative hemorrhagic strokes, and ischemic strokes. Due to the complex anatomy, the risk of these complications is higher. Transposition is an alternative technique used when decompression alone may not be sufficient. Rather than material being placed between the offending vessel and the nerve, the offending vessel is repositioned away from the trigeminal nerve and secured in a new location. Fixation may use aneurysm clips, biomedical glues, Prolene sutures, tapes, and titanium clips. Transposition may be considered when the compressing vessel is large or rigid, and decompression alone may not maintain separation over time [124,125]. These surgical methods with transposition may be long and complicated, so it is often advantageous to operate in a simpler way. It has been shown that combining decompression and transposition can achieve stable nerve relief in cases where interposition alone may not be sufficient [126]. Decompression and transposition are techniques within MVD, with Teflon placement as the standard approach in most cases and transposition reserved for unique instances in which it may be needed.

### 3.10. The Role of SRS in the Management of TN

SRS is a non-invasive treatment option for patients with TN who do not achieve adequate symptom relief from pharmacotherapy, percutaneous approaches, or MVD. SRS delivers a highly focused dose of ionizing radiation to a defined segment of the trigeminal nerve while minimizing exposure to surrounding structures. Because it does not require craniotomy or nerve manipulation, SRS is often considered for patients who are poor candidates for surgery [66]. SRS is typically performed using linear accelerator (LINAC) based systems or Gamma Knife platforms. Treatment planning relies on high-resolution imaging to identify the trigeminal nerve REZ. Accuracy is crucial to limit the collateral injury and deliver the radiation to the small nerve. Target placement closer to the brainstem and appropriately sized treatment volume have been associated with greater disruption of pain pathways, highlighting the importance of accuracy [127]. SRS generally provides delayed pain relief or recurrent symptoms and is performed without general anesthesia in most cases.

The delay may also reflect the biological effects that radiation has on nerve tissue [128]. Patients who experience recurrent symptoms after initial benefit may consider repeat SRS, and it has been shown to provide pain control in certain patients [129,130]. SRS is associated with a lower risk of significant complications than open surgical procedures. The most common effect is facial numbness, but serious complications are uncommon when proper planning, proper dosage, and accurate imaging are used. Outcomes with LINAC-based SRS are comparable to those with Gamma Knife systems [131]. While SRS is less familiar with less literature on the efficacy, it is still a treatment proven for patients who do not experience or cannot tolerate MVD for the treatment of TN.

### 3.11. Neuromodulation for Facial Pain: DBS, MCS, High Cervical SCS, Peripheral Nerve Field Stimulation

Medical therapies, percutaneous approaches (radiofrequency, GR, and balloon compression), and surgical procedures (MVD) are well-established and highly effective in treating TN [132]. While most patients experience clinically significant pain relief after one or a combination of these interventions, approximately 1–2% of patients fail to achieve a pain-free state [133]. For this subgroup, the following pain management option is neuromodulation. Neuromodulation is a technique that directly delivers electrical stimulation to specific neurologic targets to modulate neural activity, and its use is reserved for patients who are refractory to all conventional medical and surgical therapies [134,135,136]. Available techniques include deep brain stimulation (DBS), motor cortex stimulation (MCS), high cervical spinal cord stimulation (SCS), and peripheral nerve field stimulation (PNFS). Each modality differs in electrode placement, and selection depends on patient-specific factors, including anatomy, risk tolerance, and comorbidities [137].

PNFS is a minimally invasive technique involving subcutaneous implantation of electrodes in the painful trigeminal dermatome under local anesthesia. The electrodes are connected to an external generator that delivers continuous electrical stimulation [138]. Retrospective analyses demonstrated clinically meaningful responses, including reductions in pain (>50%), attack frequency, and analgesic consumption [139,140]. Although complications such as infection, electrode migration, and skin erosion may occur, they are infrequent and generally manageable [138,141]. In the short term, PNFS may provide substantial pain relief. However, a study of 15 patients demonstrated that long-term PNFS use is associated with a considerable failure rate, with a median time to failure of two years [142]. Furthermore, a meta-analysis of eleven cohort studies revealed marked heterogeneity in outcomes. In the absence of randomized controlled trials, the actual therapeutic value of PNFS in TN remains uncertain [143]. This technique should therefore be reserved for carefully selected patients.

High cervical SCS remains a salvage neuromodulatory option rather than a primary alternative to MVD with patients with classical TN and clear neurovascular compression. Its use is most appropriate in carefully selected patients with persistent refractory pain after standard pharmacologic, percutaneous, or surgical strategies have failed, are contraindicated, or poorly tolerated. High cervical SCS involves electrode placement in the epidural space of the upper cervical spinal cord, either at the C1–C2 level or at the cervicomedullary junction [144], unlike PNFS, which targets peripheral nociceptive pathways. High cervical SCS targets central pain processing pathways. Because of its deeper targets, high cervical SCS can provide broader and more diffuse analgesia. Its effectiveness has been supported by multiple case reports and retrospective studies demonstrating pain reductions exceeding 50% with minimal adverse effects [144,145,146]. At long-term follow-up of 4.4 years, patients reported a 57.1% reduction in pain and low treatment failure rates, suggesting a durable benefit [145]. Nevertheless, the scarcity of large-scale randomized controlled studies limits definitive conclusions regarding efficacy and safety [144]. Owing to its more invasive nature, high cervical SCS is generally regarded as a second-line neuromodulatory intervention following PNFS.

MCS is an invasive therapy that targets the contralateral precentral gyrus (motor cortex) corresponding to the affected trigeminal distribution through epidural or subdural electrode implantation [147]. Multiple case reports and meta-analyses have demonstrated its potential to alleviate TN-related pain. A case series of five patients who underwent MCS reported an average of 77% pain reduction at 12 months, accompanied by a substantial decrease in narcotic use [148]. A meta-analysis comparing MCS across various chronic neuropathic orofacial pain syndromes found that outcomes were significantly more favorable in patients with TN than those suffering from other pain syndromes [149]. Despite these findings, clinical responses remain variable, and long-term outcome data are limited [150]. Because MCS requires a craniectomy for electrode placement, it carries risks including infection, hematoma, and seizures [147]. Consequently, MCS is considered a third-line neuromodulatory technique option, and its use necessitates stringent patient selection.

DBS represents the final neuromodulatory option for treating refractory TN. Current literature consists of a limited number of small studies evaluating DBS in chronic neuropathic facial pain. In a survey by Ben-Haim et al., stimulation of the ventral posteromedial thalamus nucleus and the periaqueductal region resulted in meaningful pain relief in 7 patients. However, DBS is not FDA-approved for TN and is used off-label. The overall quality of evidence is low, and the technique is currently regarded as investigational [151]. Similar to MCS, DBS is highly invasive and should be reserved exclusively for last-resort management.

Overall, neuromodulation therapies have emerged as promising adjunctive options for patients with chronic TN refractory to pharmacological and surgical interventions. Evidence from case reports, retrospective studies, and meta-analyses suggests that these techniques can significantly improve pain control while maintaining acceptable safety profiles. Nonetheless, the absence of large-scale randomized controlled trials precludes definitive determination of optimal patient selection and long-term efficacy. High-quality prospective research is therefore essential to better define the role of the four neuromodulation techniques and to support their broader clinical integration.

### 3.12. Management of Refractory TN

TN is a highly debilitating condition that substantially impairs one’s quality of life. When a patient fails to respond to, or experiences adverse effects from, current standard approaches, including pharmacological therapies, neurosurgical procedures, percutaneous interventions, and neuromodulatory techniques, the condition is referred to as refractory TN [152].

Management of patients experiencing persistent, severe, treatment-resistant TN is challenging. Due to monotherapy’s failure to provide an adequate response, providers must consider multimodal interventions combining several pharmacological agents and/or surgical strategies. This method allows for dose reduction, thereby minimizing side effects while enhancing both the duration and magnitude of treatment benefit [153]. A 2017 meta-analysis, for instance, demonstrated that a drug-focused therapy including sumatriptan, intranasal lidocaine, intravenous lidocaine, and BTX, and an intervention-focused therapy combining continuous and pulsed RFA, provide effective pain reduction in refractory TN cases [154]. In a separate review, Pergolizzi Jr et al. suggested the potential efficacy of integrating third-generation anticonvulsants, new calcitonin gene-related peptide blockers, ketamine, and cannabinoids with first-line agents such as carbamazepine and oxcarbazepine [155]. However, because of the paucity of research exploring multimodal approaches to refractory TN, no standardized management protocol currently exists. Furthermore, the exact combination of therapies depends on patients’ treatment response, comorbidities, and pain etiology and characteristics [133,153,156]. As care plans are highly individualized, providers’ clinical experience is essential.

In addition to combination therapy, another possible approach for refractory TN patients is to repeat interventions that offer short-term relief, with the expectation that subsequent treatments may extend the duration of pain improvement. Sequential administration of therapies may provide continuous pain relief without patients experiencing a complete return to baseline pain levels. For example, BTX injections are known to provide pain reduction for approximately three to six months, particularly when used as an adjunct to other pharmacological therapies [157,158]. Multiple studies have consistently demonstrated that repeated injections prolong the duration of relief as the effects of prior injections begin to diminish [157,158,159]. Similar findings have been reported for procedures such as RFA, balloon compression, and SRS without an increase in adverse events [97,160,161]. However, each procedure carries inherent risks, requiring providers to have a thorough understanding of each patient’s profile.

Overall, an essential factor in the management of failed TN patients is multidisciplinary care. To optimize quality of life and address all aspects of the disorder, collaboration among primary care providers, pain physicians, neurologists, and psychological specialists is essential [162]. The combined efforts of these professionals facilitate the delivery of comprehensive, individualized care.

## 4. Hemifacial Spasm (HFS)

### 4.1. Diagnosis and Non-Surgical Management in HFS

HFS is a neurological disorder characterized by paroxysmal, involuntary twitching of the facial muscles on one side of the face, caused by the ipsilateral facial nerve (the seventh CN). Bilateral involvement occurs in fewer than 5% of cases. It is a rare condition with a prevalence of 14.5 per 100,000 in women and 7.4 per 100,000 in men, indicating that it is almost twice as likely to occur in women as in men. The most common cause of HFS is compression of the facial nerve as it exits the brainstem by an ectatic or aberrant artery [163]. Chronic vascular contact is associated with abnormal facial nerve activity, resulting in involuntary muscle contractions. Secondary causes, including tumors, are less common but should be considered during evaluation.

Diagnosis of HFS is primarily clinical, but MRI is used to evaluate the facial nerve and identify sites of compression [164]. The current first-line non-surgical treatment for HFS is BTX injections, which is widely accepted as an effective therapy. BTX acts by inhibiting acetylcholine release at the neuromuscular junction, resulting in a temporary reduction in muscle contractions [164,165]. Studies have demonstrated significant improvement in facial spasms in the majority of patients. However, the therapeutic effect of BTX is temporary, with an average duration of approximately 12 weeks, necessitating repeat injections for ongoing symptom management [166]. For patients who are unable to tolerate BTX injections, oral medications may be considered. Agents such as carbamazepine, baclofen, pizotifen, and clonazepam may be used, but they are often associated with undesirable side effects. Gabapentin or levetiracetam may be better tolerated in the elderly population [165].

HFS can significantly impair quality of life due to its visible nature and functional impact. While non-surgical treatments such as BTX provide effective symptom control, ongoing monitoring is required because of the need for repeat procedures. The current lack of literature emphasizes the need for continued research into alternative treatment options for patients who are not candidates for MVD [167]. MVD remains the standard definitive treatment for HFS, while BTX and oral medications serve as valuable alternatives for patients who are elderly or unsuitable for surgical intervention.

### 4.2. MVD for HFS: Surgical Pearls

MVD is considered the definitive surgical treatment for HFS. The goal of the procedure is to relieve neurovascular compression of the facial nerve at its root exit zone without causing neural injury. MVD is performed under general anesthesia using a posterior approach, most commonly via a retrosigmoid craniectomy. The craniectomy is typically positioned just inferior to the ipsilateral transverse sinus, and once the offending vessel is identified, it is carefully mobilized away from the facial nerve. A teflon patch is then interposed between the vessel and nerve to maintain separation and minimize the risk of recurrent compression [168,169,170].

Intraoperative neuromonitoring is recommended to optimize surgical outcomes and prevent injury to adjacent neural structures, such as the vestibulocochlear nerve, which lies in proximity to the facial nerve [169]. Recurrence of HFS following MVD has been associated with several factors, including excessive teflon placement, arachnoid adhesions, and formation of teflon granulomas. Excessive or improperly positioned Teflon may lead to persistent or recurrent neurovascular compression, while arachnoid adhesions can contribute to recompression of the facial nerve over time. Additionally, Teflon granuloma formation represents a delayed inflammatory complication that may result in symptom recurrence. Avoiding blood contamination of the teflon during the procedure is an essential strategy in preventing granuloma formation [170].

While preoperative MRI is routinely used to evaluate neurovascular compression, it may not always be apparent, and MVD has nevertheless been shown to be effective in select patients [171]. In such cases, intraoperative exploration may reveal subtle compression not detected on preoperative imaging. In patients with recurrent symptoms after an initial MVD, a repeat procedure may be considered following careful reassessment of the compressing vessel [170]. Overall, MVD remains an established and highly effective surgical option for HFS when non-surgical treatments fail.

## 5. Glossopharyngeal Neuralgia (GN)

### 5.1. Diagnosis and Non-Surgical Management in GN

GN is a rare CN disorder characterized by electric, shooting pain in the sensory distribution of the auricular and pharyngeal branches of the glossopharyngeal (IX) and vagus (X) CNs. The affected areas include the ear, base of the tongue, throat, tonsillar fossa, and angle of the jaw. It is easily triggered by talking, swallowing, and coughing, causing brief unilateral pain [32].

Diagnosis of GN is primarily clinical and is based on the characteristic pain location and triggers. Because symptoms may mimic dental conditions, initial imaging, such as orthopantomograms and PA skull radiographs, is used to rule out a dental cause of pain. Diagnostic confirmation may be obtained with a glossopharyngeal nerve block using the intraoral anterior tonsillar pillar technique, providing temporary pain relief [172]. The use of the block is both a confirmatory block and a temporary alleviation of pain [173].

**Figure 3:**
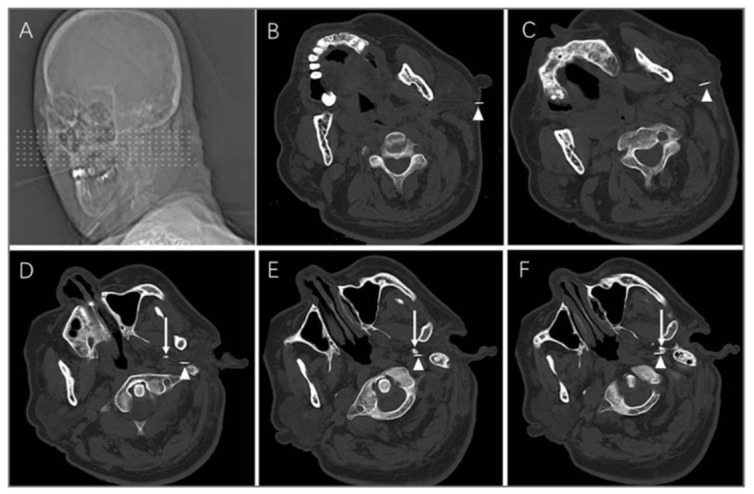
(**A**) Puncture site for glossopharyngeal nerve access. (**B**–**F**) Sequential CT images demonstrating advancement of the needle to the medial margin of the styloid process. White arrows indicate the needle, and white triangles indicate the styloid process. (Reprinted/adapted with permission from Ref. [176] Jia et al. 2020. This figure is published under the Creative Commons Attribution–NonCommercial (CC BY-NC) License, which permits noncommercial use, distribution, and reproduction in any medium, provided the original authors and source are credited).

Conservative management for patients with GN typically begins with anticonvulsant medications, reflecting the similarities in pain mechanisms between GN and TN. Carbamazepine is generally preferred over phenytoin and is considered first-line therapy for symptom control. Both GN and TN have been shown to respond to sodium-channel blocking agents [32]. Patients who are unable to tolerate carbamazepine due to its side effects may be given levetiracetam, which is effective for neuropathic pain [174]. In patients with an inadequate response to medications, radiofrequency-based techniques have also been reported in the management of GN. Pulsed radiofrequency treatment targets the glossopharyngeal nerve to disrupt pain transmission and has been shown to provide both short-term and sustained pain relief [175,176]. The use of image guidance, including CT or ultrasound, allows for precise needle placement and lowers the risk of complications (Figure 3) [176]. There are many conservative and non-surgical treatments for GN; however, further research is needed to evaluate additional therapies given the condition’s rarity.

### 5.2. MVD for GN: Surgical Pearls

MVD is considered the standard surgical treatment for patients with GN who do not achieve adequate pain relief with pharmacological therapy or minimally invasive procedures. MVD for GN involves the identification of the offending vessel compressing the glossopharyngeal nerve, most commonly at the REZ. Once the vessel is identified, it is mobilized away from the nerve, and Teflon is used to maintain separation and prevent recurrence. Different decompression strategies may be required depending on vessel mobility. In cases where the offending vessel cannot be repositioned using the standard Teflon interposition technique, a fibrin glue-coated Teflon sling may be used to secure the vessel [177].

**Figure 4:**
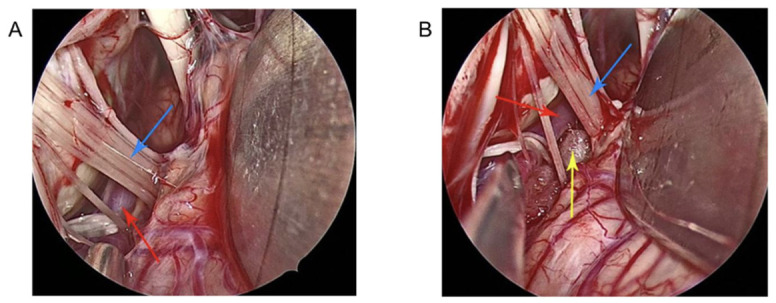
(**A**) Endoscopic view demonstrating the left vertebral artery (red arrow) and left glossopharyngeal nerve (blue arrow). (**B**) Placement of a Teflon pad (yellow arrow) between the left vertebral artery and glossopharyngeal nerve. (Reprinted/adapted with permission from Ref. [180] Jiang et al. 2023. Published under the Creative Commons Attribution (CC BY) License, which permits unrestricted use, distribution, and reproduction in any medium, provided the original authors and source are credited).

MVD without neurectomy has been shown to provide effective pain relief with complete pain resolution in the majority of patients [178]. The microasterional approach allows access to the lower CNs with limited bone removal and has been used to achieve adequate exposure without fixed cerebellar retractors [179]. Endoscopic-assisted and fully endoscopic MVD techniques have also been successful. Endoscopic visualization provides improved illumination, wider viewing angles, and enhanced visualization compared with traditional microscopic approaches. This more comprehensive view reduces the risk of complications (Figure 4) [180].

Another technique using a combined transcondylar fossa and unilateral transcerebellomedullary fissure approach, with fixation of the offending vessel to the dura mater, has been reported to help prevent recurrence. This technique allows for wider exposure of the lower CPA, reduced cerebellar retraction, improved visualization, and minimal manipulation of lower CNs. It has also achieved favorable outcomes with low complication rates [181]. Compression directly involving the glossopharyngeal nerve REZ has been associated with better long-term outcomes. In contrast, lower degrees of compression have been correlated with shorter pain-free intervals before recurrence [182]. Revision or alternative decompression strategies may be considered in cases of persistent or recurrent pain, particularly when initial decompression is limited by vessel anatomy or incomplete separation. Given the rarity of the condition, further research into MVD techniques for the treatment of GN is needed to improve surgical efficiency and optimize long-term pain control.

### 5.3. SRS for GN

MVD remains the standard surgical treatment for GN; however, because the condition commonly affects older patients, SRS has been explored as a noninvasive alternative for patients who are poor candidates for MVD. SRS for GN delivers a focused dose of ionizing radiation to a segment of the glossopharyngeal nerve, aiming to reduce pain transmission. The most commonly reported radiosurgical target is the glossopharyngeal meatus, which allows precise targeting of the nerve as it exits the skull base [183,184]. Accurate targeting of the glossopharyngeal nerve relies on thin-slice MRI and bone-based CT imaging for optimal visualization. The combination of MRI and CT is considered essential for safe and effective treatment planning [185]. Due to the rarity of GN, the optimal radiation dose and long-term outcomes remain uncertain [186].

Patients who experience pain recurrence after initial SRS may benefit from repeat treatment. Repeat procedures have been found to provide additional pain relief and are generally well tolerated [187,188]. Because SRS does not involve direct manipulation of the nerve, it is considered a reasonable option for patients at high surgical risk [189]. SRS is generally associated with a low risk of serious complications. Reported adverse effects are infrequent and typically mild. Patients often demonstrate improvement in pain-related functional limitations, including eating, swallowing, speaking, and drinking [183]. However, the limited prevalence of GN has constrained both the size of available studies and the availability of long-term outcome data. Systematic reviews emphasize the need for larger, multicenter studies with extended follow-up to define better treatment durability, optimal targeting strategies, and dosing parameters [187]. As a result, SRS is generally considered a secondary or alternative treatment rather than a replacement for surgical decompression.

## 6. Rare Neurovascular Compression Syndromes

### 6.1. IV—Superior Oblique Myokymia

Superior oblique myokymia (SOM) is a rare ocular motor disorder characterized by brief, intermittent, involuntary contractions of the superior oblique muscle, resulting in visual disturbance. The condition has been associated with vascular compression of the trochlear nerve (fourth CN); however, this compression is often complicated to visualize on MRI [180,181,182,183,184,185,186,187,188,189,190,191,192]. The rarity of SOM has led to a limited understanding of its etiology, and recognition of the neurovascular compression mechanism emerged only in later decades [193]. The symptoms of SOM include double vision or rotating images, known as oscillopsia. Unlike other causes of oscillopsia, SOM is unilateral, episodic, and involves very small, low-amplitude movements confined to the superior oblique muscle.

Initial management of SOM typically involves conservative pharmacological therapy. Anticonvulsants such as carbamazepine have been reported to provide symptomatic benefit with minimal adverse effects [194]. In patients with refractory symptoms, MVD at the brainstem exit zone of the trochlear nerve has been reported as a surgical option with favorable outcomes following decompression [191,195]. Due to the low prevalence of SOM, long-term treatment outcomes and standardized management strategies remain limited, and further investigation is required for patients who do not respond to pharmacotherapy or cannot tolerate surgical intervention.

### 6.2. VI—MVD for Abducens Nerve Palsy

Abducens nerve palsy results from dysfunction of the sixth CN and presents with impaired ipsilateral eye abduction. It is the most common isolated CN palsy in adults and the second most common in children. In adults, abducens nerve palsy may be categorized as vasculopathic or nonvasculopathic, with the former occurring more frequently in older patients and in those with conditions such as diabetes [196].

Neurovascular compression of the abducens nerve has been identified as a potential etiology in select cases. When imaging confirms neurovascular conflict, careful monitoring of symptoms may prompt treatment, and MVD may be considered in select cases. Some cases with isolated abducens nerve palsy, presumably due to AICA compression, may indicate idiopathic causes, and MVD may not be the best treatment option [197,198]. Surgical decompression via a retrosigmoid approach has been reported to result in symptomatic improvement in patients with confirmed compression. Because abducens nerve compression syndromes are rare and the available literature remains limited, current treatment recommendations are primarily based on small case series and case reports [199,200]. While MVD has demonstrated effectiveness in carefully selected patients, alternative treatment options remain limited for those who are not suitable candidates for surgery.

### 6.3. VII—Nervus Intermedius Neuralgia

Nervus intermedius neuralgia (NIN) is characterized by paroxysmal, sharp pain deep within the auditory canal. The condition is attributed to compression of the nervus intermedius (the seventh CN), a component of the facial nerve complex. It has clinical overlap with both TN and GN, which complicates diagnosis and treatment [201].

Accurate diagnosis relies on MRI to evaluate neurovascular compression of the facial nerve complex. Although MVD is an established treatment for TN, there is limited evidence supporting its efficacy for NIN [202]. Nevertheless, surgical series and case reports have documented symptom improvement following MVD in patients with refractory NIN [203,204]. Nonsurgical options have been explored. A fluoroscopy-guided anesthetic block targeting the geniculate branch of the facial nerve has been reported to provide short-term pain relief [202]. Although treatment strategies are often extrapolated from those used for TN and GN, there remains insufficient evidence to support a standardized treatment approach [205]. Precise diagnosis is essential for appropriate management of NIN, particularly given the limited literature and the lack of established alternative therapeutic options.

### 6.4. VIII—Surgical Management of Vestibulocochlear Compression Syndrome

Neurovascular compression of the vestibulocochlear nerve (eighth CN) has been associated with tinnitus and vertigo. Vascular contact between the nerve and adjacent arteries may result in persistent auditory and vestibular symptoms. MRI is essential for identifying neurovascular conflict and excluding alternative etiologies [206].

MVD has been reported as a surgical treatment option for vestibulocochlear compression syndrome and has been shown to improve or resolve tinnitus and vertigo following decompression [207,208,209,210]. Patients with severe, disabling, or intractable vertigo are the best candidates for surgery with the supporting imaging of the neurovascular compression of CN VIII. Additional studies have supported the efficacy of MVD in patients with refractory symptoms after conservative management [209,210]. Although surgical outcomes appear favorable in selected cases, vestibulocochlear compression syndromes remain uncommon, and careful patient selection is critical. Continued investigation is necessary to better define diagnostic criteria, predictors of treatment response, and long-term outcomes following surgical intervention.

### 6.5. X—Vagus Neuralgia, HELPS, Vancouver Syndrome

The vagus nerve (tenth CN) has motor, sensory, and autonomic functions. Vascular compression of the vagus nerve has been associated with two clinical conditions: hemilaryngopharyngeal spasm (HELPS) and vagus-associated neurogenic cough caused by unilateral vascular encroachment at the nerve root, also known as Vancouver syndrome [211]. HELPS is characterized by paroxysmal, unilateral contractions of the laryngeal and pharyngeal musculature, often presenting as throat tightening or choking sensations and coughing. In contrast, Vancouver syndrome presents as a chronic, nonproductive cough attributed to neurovascular irritation of the vagus nerve. With the rarity of these conditions, the diagnostic and pathophysiologic classification is still evolving.

**Figure 5:**
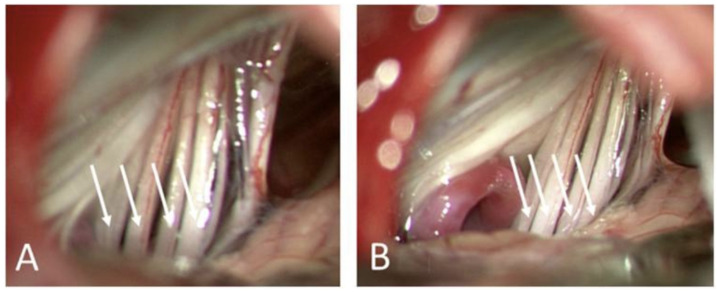
Microscopic view of four rootlets of the vagus nerve. (**A**) Pre-decompression image demonstrating a loop of the posterior inferior cerebellar artery compressing the nerve anteriorly and displacing it posteriorly. (**B**) Post-decompression image following mobilization of the vascular loop. Arrows indicate the site of neurovascular compression and subsequent decompression. (Reprinted/adapted with permission from Ref. [211] Honey et al. 2025. Published under the Creative Commons Attribution (CC BY) License, which permits unrestricted use, distribution, and reproduction in any medium, provided the original authors and source are credited).

Both conditions can be difficult to diagnose; however, an MRI may help identify the offending vessel responsible for the compression. Initial management includes pharmacologic therapy. Nifedipine and antiepileptic medications have been used for HELPS, while carbamazepine may be used for Vancouver syndrome [212]. BTX injections may reduce symptoms in HELPS, but results have been inconsistent. BTX has been shown to reduce spasms without fully resolving symptoms, whereas MVD has demonstrated complete symptom resolution in reported cases [213,214]. For Vancouver syndrome, MVD has likewise been associated with complete symptom resolution following identification of the offending vessel (Figure 5) [211].

Although it is rare, hypertension associated with vascular compression of the vagus nerve is important to consider. Compression of the vagus nerve at the REZ in the brainstem, often by the PICA, can lead to neurogenic hypertension as a result of impaired parasympathetic tone [215]. Reduced vagal inhibition leads to unopposed sympathetic activity and corresponding severe or persistent hypertension. Additionally, the vagus nerve is crucial for transmitting signals from aortic baroreceptors to the nucleus tractus solitarius (NTS) [212,216]. Compression disrupts this, destroying the buffer system that regulates blood pressure, resulting in neurogenic hypertension.

### 6.6. Brain Stem Compression Syndrome and MVD

In sporadic cases, large ectatic arteries, such as the vertebral or basilar arteries, may compress the brainstem, leading to symptoms of pontine or medullary dysfunction, including hemiparesis, dysphagia, or respiratory distress [217]. In the limited available literature, MVD has been reported as a surgical option aimed at relieving brainstem compression through vascular mobilization and has demonstrated satisfactory neurological improvement following decompression [218].

When symptoms are directly attributable to mechanical compression, MVD has been shown to result in symptom resolution or significant clinical improvement [219,220]. Intraoperative electrophysiologic monitoring has been emphasized as an essential adjunct to reduce the risk of neural injury during brainstem decompression procedures; however, not all patients experience sustained symptom relief [220,221]. Given the rarity of brainstem compression syndrome, further investigation is needed to define better patient selection criteria, optimal surgical techniques, and long-term outcomes. A summary of the rare NVCS is displayed in Table 1.

## 7. Conclusions

NVCS are challenging disorders that sit at the intersection of detailed neuroanatomy, vascular pathology, and complex pain physiology. As outlined in this review, successful management depends on accurate diagnosis, thoughtful imaging interpretation, and a stepwise, individualized treatment approach. Options range from pharmacologic therapy to percutaneous procedures, MVD, SRS, and neuromodulation, with each playing a role depending on the patient’s presentation and goals of care. However, the strength of evidence supporting these approaches varies considerably across syndromes. While more common conditions such as TN are supported by robust clinical data, many of the rarer NVCS discussed are informed by case reports and small series trials, and lack standardized management guidelines. Advances in imaging and surgical visualization continue to improve precision and safety, but careful patient selection and multidisciplinary collaboration remain central to achieving durable symptom relief. Ongoing research is necessary to refine treatment algorithms and strengthen the evidence for both common and rare NVCS.

## Figures and Tables

**Table 1:** Summary of rare cranial nerve compression syndromes.

Syndrome	Clinical Manifestations	Typical Offending Vessels	First-Line Treatment Options
Superior Oblique Myokymia	Episodic, unilateral double vision or rotating images (oscillopsia)	SCA, PCA	Carbamazepine and other anticonvulsants, MVD
Abducens Nerve Palsy	Impaired ipsilateral eye abduction	AICA	Observation/monitoring, MVD
Nervus Intermedius Neuralgia	Paroxysmal sharp deep ear pain	AICA	Nerve block, MVD
Vestibulocochlear Compression Syndrome	Tinnitus, vertigo	AICA	MVD
Vagus Neuralgia	Hemilaryngopharyngeal spasm (HELPS): throat tightening, choking sensation, coughing Vancouver syndrome: chronic nonproductive cough	PICA	HELPS: nifedipine and antiepileptic medications, BTX injections, MVD Vancouver syndrome: carbamazepine, MVD
Brainstem Compression Syndrome	Hemiparesis, dysphagia, and respiratory distress	Vertebral artery, basilar artery	MVD

## Data Availability

No new data were created or analyzed in this study. Data sharing is not applicable to this article.

## Abbreviations

The following abbreviations are used in this manuscript:

NVCS	Neurovascular compression syndromes
CN	Cranial nerves
REZ	Root entry zone
TN	Trigeminal neuralgia
HFS	Hemifacial spasm
GN	Glossopharyngeal neuralgia
MVD	Microvascular decompression
SRS	Stereotactic radiosurgery
MRI	Magnetic resonance imaging
VR	Virtual reality
BTX	Botulinum toxin
CPA	Cerebellopontine angle
CSF	Cerebrospinal fluid
AICA	Anterior inferior cerebellar artery
SCA	Superior cerebellar artery
PICA	Posterior inferior cerebellar artery
CNS	Central nervous system
PNS	Peripheral nervous system
MS	Multiple sclerosis
SLE	Systemic lupus erythematosus
CISS	Constructive interference in steady states
FIESTA	Fast Imaging Employing Steady State Acquisition
CT	Computed tomography
3D TOF MRA	3D Time-of-Flight Magnetic Resonance Angiography
NMUS	Neuromuscular ultrasound
DTI	Diffusion tensor imaging
HC	Hemicrania Continua
MPDS	Myofascial pain dysfunction syndrome
HDRS	Hamilton Depression Rating Scale
RFR	Radiofrequency rhizotomy
GR	Glycerol rhizotomy
PBC	Percutaneous balloon compression
LINAC	Linear accelerator
DBS	Deep-brain stimulation
MCS	Motor cortex stimulation
SCS	Spinal cord stimulation
PNFS	Peripheral nerve field stimulation
SOM	Superior oblique myokymia
NIN	Nervus intermedius neuralgia
HELPS	Hemilaryngopharyngeal spasm
NTS	Nucleus tractus solitarius
